# Investigation of the Physiological and Biochemical Effects of Brassinosteroid and Nitric Oxide in Two Wheat Varieties with Different Drought Tolerance

**DOI:** 10.3390/plants15213312

**Published:** 2026-10-26

**Authors:** Eda Günay, Okan Acar

**Affiliations:** Department of Biology, Faculty of Science, Çanakkale Onsekiz Mart University, 17100 Çanakkale, Türkiye; oacar@comu.edu.tr

**Keywords:** drought stress, wheat, nitric oxide, brassinosteroid, oxidative stress, antioxidant enzymes, reactive oxygen species, *Triticum aestivum* L.

## Abstract

Drought, which causes oxidative stress by increasing the amount of reactive oxygen species (ROS) in plants, leads to a decrease in yield in bread wheat (*Triticum aestivum* L.). On the other hand, nitric oxide (NO) and brassinosteroids (BRs) are known to maintain cellular homeostasis under drought stress in wheat through various signal transduction pathways. This research focuses on the individual and combined effects of NO (Sodium nitroprusside, SNP) and BR (Epibrassinolide, EBL) on physiological and biochemical parameters in two wheat varieties with different drought tolerances: under polyethylene glycol (PEG)-induced and water-scarcity-induced drought stress. Drought stress decreased ascorbate peroxidase (APX), glutathione reductase (GR), and peroxidase (POX) activities in the drought-sensitive Kenanbey variety, while increasing superoxide dismutase (SOD), GR, APX, and CAT activities in the tolerant NKÜ Lider variety. Exogenous SNP and EBL treatments effectively mitigated drought-induced membrane damage and oxidative stress, leading to significant reductions in electrolyte leakage (EL), lipid peroxidation (TBARS), and ROS accumulation in both varieties. In the NKÜ Lider, under both PEG-induced osmotic stress and water-scarcity conditions, the combined SNP and EBL treatment increased root length (RL), biomass, dry weight (DW), and SOD isoenzyme band intensity, while reducing electrolyte leakage (EL), compared with the individual treatments. In the Kenanbey variety, under water-scarcity conditions, the combined treatment improved biomass, SPAD values, CAT and POX activities, and SOD isoenzyme band intensity, while reducing EL. Additionally, the dramatically reduced SPAD and water content in both drought treatments were increased to control plant levels with individual and combined treatments of SNP and EBL. These findings highlight the potential of NO and BR treatments as promising strategies to improve physiological and biochemical responses associated with drought tolerance in wheat varieties. These findings provide experimental evidence for the development of foliar regulators for drought tolerance in wheat seedlings.

## 1. Introduction

Climate change, global temperature increase, and water scarcity are among the most important environmental problems threatening agricultural production. Drought stress is considered one of the abiotic stress factors that has the most negative impact on plant growth and yield [1]. Drought causes a decrease in water potential, disruption of cell turgor, reduction in photosynthetic capacity, and the suppression of metabolic processes in plants. As a result, plant growth, development, and crop yield decrease significantly. With the increasing effects of global climate change, it is predicted that drought-induced agricultural losses will become even more severe in the future [2].

Wheat (*Triticum aestivum* L.), along with rice and maize, is one of the staple food sources worldwide and a strategic cereal crop with high adaptability [3]. However, wheat production is severely negatively affected by environmental stress conditions such as drought and high temperatures [4]. Furthermore, drought reducing wheat yields poses a significant threat to global food security. Indeed, the increase in food demand due to global population growth has made it imperative to develop sustainable strategies for breeding drought-tolerant and high-yielding wheat varieties [5].

One of the most important consequences of reduced cellular water content in plants is the formation of reactive oxygen species (ROS) as a result of the misdirection of electrons in photosynthesis and respiration pathways. The severity and duration of drought stress cause excessive increases in ROS concentrations. Excessive accumulation of ROS such as superoxide radical (O_2_^•−^), hydrogen peroxide (H_2_O_2_), hydroxyl radical (^•^OH) and singlet oxygen (^1^O_2_) leads to increased oxidative damage at the cellular level [6]. Accordingly, peroxidation in membrane lipids, protein oxidation, pigment loss and enzyme inhibition increase the permeability of the cell membrane and cause damage at the cellular level in plants [7].

Reactive nitrogen species (RNS) consist of highly reactive molecules such as nitric oxide (NO), peroxynitrite (ONOO^−^), nitrogen dioxide (NO_2_), nitrosonium cation (N^++^), and dinitrogen trioxide (N_2_O_3_) [8]. While RNS act as signaling molecules in cells at low concentrations, their accumulation at high concentrations causes nitrosative stress. In particular, the reaction of NO with O_2_^•−^ to form ONOO^−^ can increase the severity of cellular damage. However, controlled NO production plays important roles in regulating the antioxidant defense system and enhancing stress tolerance [9].

Plants can mitigate the harmful effects of oxidative and nitrosative stress through enzymatic and non-enzymatic antioxidant defense systems [10]. Antioxidant enzymes such as superoxide dismutase (SOD), catalase (CAT), ascorbate peroxidase (APX), glutathione reductase (GR), and peroxidase (POX) play a fundamental role in the detoxification of ROS. While SOD converts superoxide radicals into H_2_O_2_, enzymes such as CAT, APX, and POX catalyze the conversion of H_2_O_2_ into harmless water [11]. This contributes to the maintenance of intracellular redox homeostasis. However, if the severity of the stress increases, an insufficient antioxidant capacity leads to increased oxidative damage [12].

In recent years, studies aimed at increasing stress tolerance in plants have focused on signaling molecules and plant growth regulators. Among these molecules, nitric oxide (NO) acts as an important signaling molecule in plants and plays a role in regulating defense mechanisms under stress conditions [13]. NO is involved in various physiological processes such as stomatal closure, regulation of ion homeostasis, enhancement of antioxidant enzyme activities, and maintenance of cellular redox balance [14]. Furthermore, it has been reported that NO increases plant tolerance to stress conditions by interacting with ROS and phytohormones [15]. In particular, many studies have shown that NO donors such as sodium nitroprusside (SNP) exhibit protective effects against drought stress [16].

One of the important groups of phytohormones that enhance stress tolerance in plants is brassinosteroids (BRs). BRs are involved in numerous physiological processes, including cell division, plant elongation, photosynthesis, senescence, and stress responses [17]. In particular, 24-epibrassinolide (EBL) strengthens the antioxidant defense system by maintaining membrane stability under environmental stress conditions and contributes to the preservation of photosynthetic capacity [18]. BR treatments have been reported to reduce oxidative damage induced by drought stress and improve plant growth in various plant species [19]. However, the mechanisms underlying the interactions of BRs with other signaling molecules have not yet been fully elucidated [20].

Evidence from pepper plants indicates that BR-induced NO production contributes to antioxidant defense and improved tolerance under water stress [21]. More recent molecular evidence has further demonstrated crosstalk between NO and BR signaling pathways in the regulation of plant growth and development [22]. However, whether this interaction contributes to drought responses in wheat, particularly when NO and BR are supplied together, remains insufficiently understood.

The independent effects of NO and BRs on drought stress have been investigated in numerous studies. However, there is a lack of data regarding the physiological and biochemical responses of combined treatments of these two molecules in wheat under drought stress. In this context, the study focuses on investigating the physiological and biochemical effects of individual and combined treatments of SNP and EBL on drought tolerance in the drought-tolerant NKÜ Lider and drought-sensitive Kenanbey varieties.

Plant responses to drought depend on genotype as well as the nature and severity of water deficit. Polyethylene glycol (PEG)-6000 is commonly used to induce osmotic stress under controlled conditions, allowing water availability to be reduced in a relatively controlled manner [23]. In contrast, withholding irrigation allows water deficit to develop progressively as the growing substrate dries, thereby simulating natural field water deficit and providing a complementary approach to investigating plant responses to declining water availability. In the present study, 15% (*w*/*v*) PEG-6000 was used to represent moderate osmotic stress under controlled conditions, whereas water withholding was employed to simulate progressive water scarcity, potentially representing a more severe water deficit scenario. Because the severity of water deficit was not independently quantified, these treatments were considered contrasting experimental approaches rather than definitively established levels of drought severity. Their comparison enabled us to investigate whether the physiological and biochemical responses of drought-tolerant and drought-sensitive wheat varieties, as well as their responses to SNP and EBL, differed between controlled osmotic stress and water scarcity.

Although the individual roles of NO and BR in plant responses to drought stress have been investigated in previous studies, the physiological and biochemical effects of their combined treatment in wheat under drought stress remain insufficiently understood. Therefore, this study investigated the effects of individual and combined treatments with SNP and EBL on drought responses in two bread wheat varieties with contrasting drought tolerance, NKÜ Lider and Kenanbey.

We hypothesized that (i) foliar pre-treatment with SNP and EBL could help alleviate drought-induced growth inhibition and oxidative damage in wheat seedlings; (ii) combined SNP + EBL treatment could have complementary effects on antioxidant defense compared with individual treatments, although the magnitude of these effects might vary depending on the parameter examined; and (iii) physiological and biochemical responses to these treatments could differ between NKÜ Lider and Kenanbey and between PEG-induced osmotic stress and water scarcity. To examine these hypotheses, we assessed growth-related traits, plant water status, cellular damage, and antioxidant defense, including key antioxidant enzyme activities. By evaluating these responses, this study aimed to clarify the potential contributions of SNP and EBL to wheat responses under drought stress and provide further insights into the physiological and biochemical processes associated with drought tolerance.

## 2. Results

### 2.1. Effects of Drought Stress on Growth Parameters

**Figure 1:**
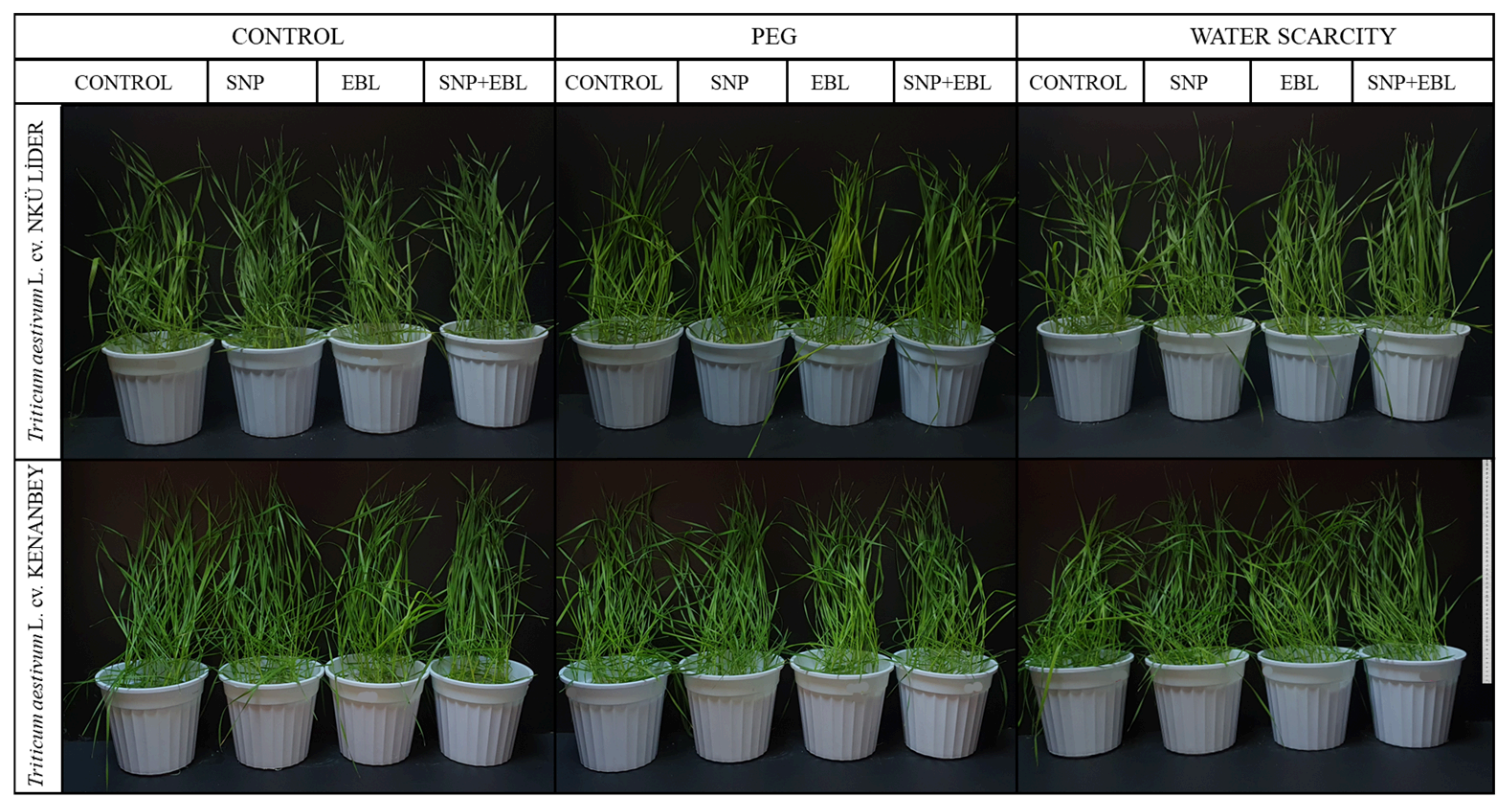
Effects of SNP and EBL treatments under drought stress on seedling development of NKÜ Lider and Kenanbey wheat varieties.

**Figure 2:**
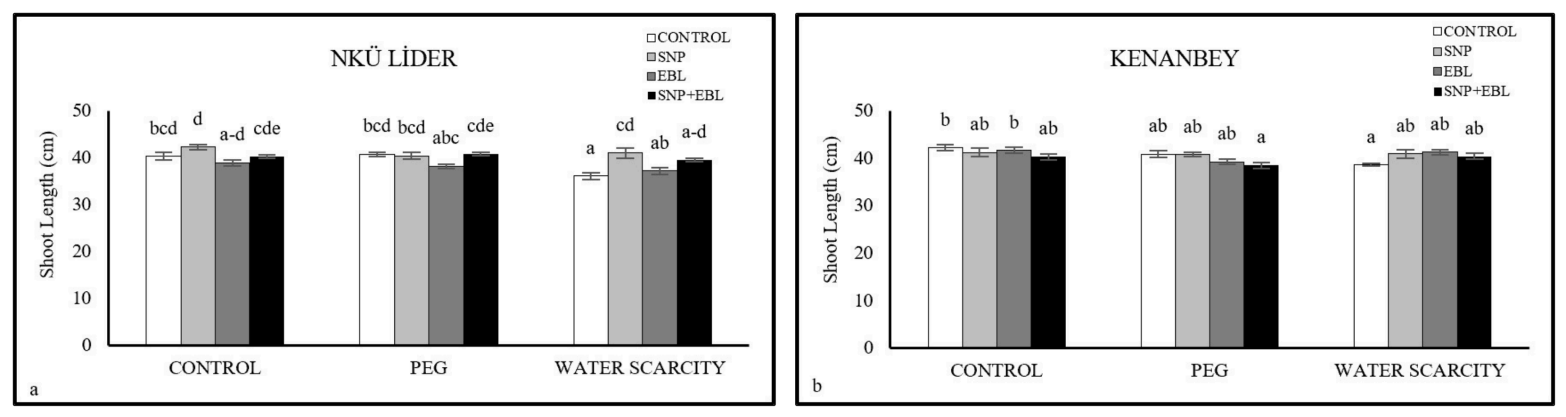
Effects of SNP and EBL treatments on shoot length in the NKÜ Lider (**a**) and Kenanbey (**b**) under drought stress. Letters indicate statistically significant differences (*p* < 0.05).

**Figure 3:**
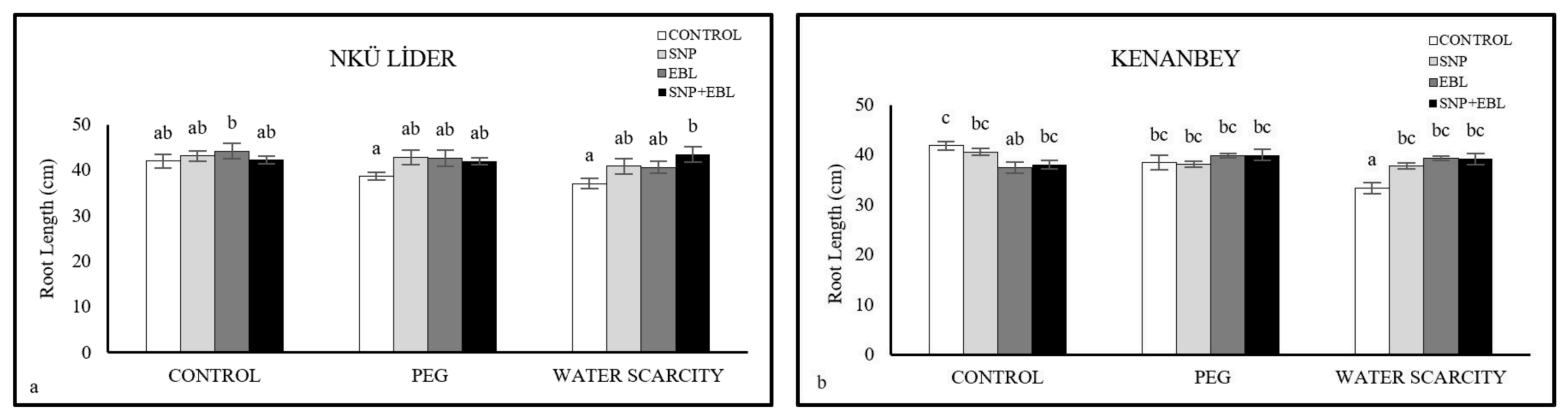
Effects of SNP and EBL treatments on root length in NKÜ Lider (**a**) and Kenanbey (**b**) varieties under drought stress. Letters indicate statistically significant differences (*p* < 0.05).

**Figure 4:**
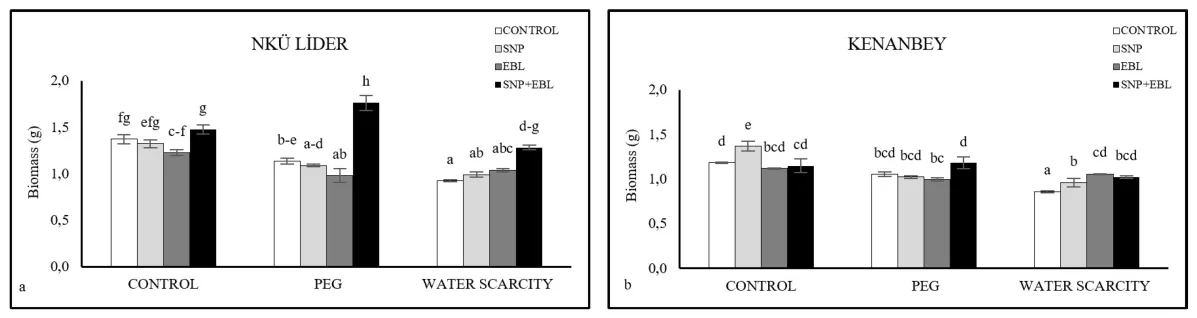
Effects of SNP and EBL treatments on biomass in NKÜ Lider (**a**) and Kenanbey (**b**) varieties under drought stress. Letters indicate statistically significant differences (*p* < 0.05).

**Figure 5:**
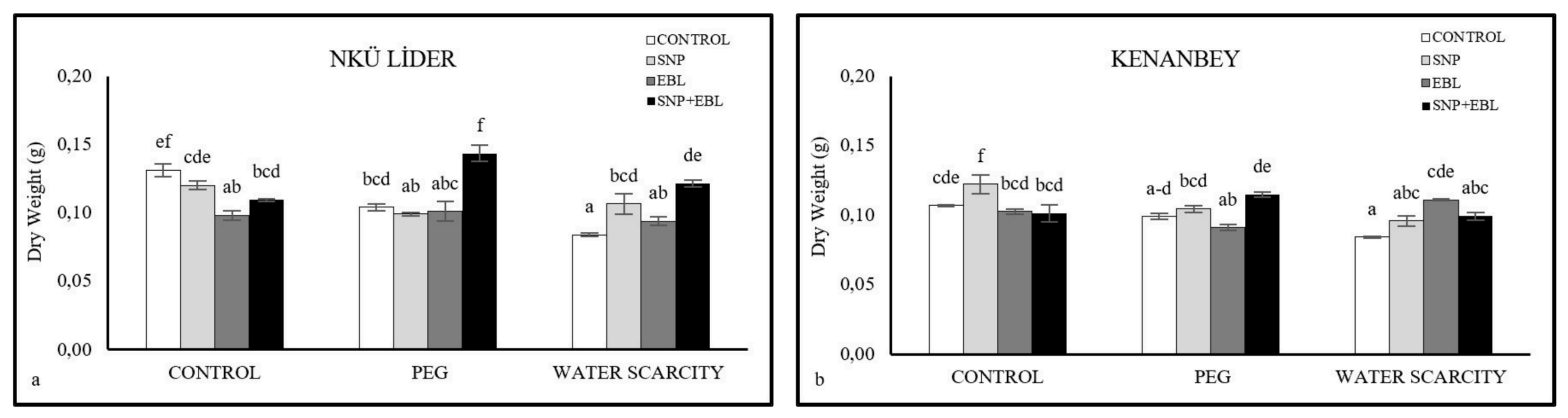
Effects of SNP and EBL treatments on dry weight of NKÜ Lider (**a**) and Kenanbey (**b**) varieties under drought stress. Letters indicate statistically significant differences (*p* < 0.05).

Drought stress suppressed seedling development in the NKÜ Lider and Kenanbey wheat varieties (Figure 1). The effects of drought stress on shoot length, root length, biomass, and dry weight are presented separately in the corresponding figures and are described below (Figure 2, Figure 3, Figure 4 and Figure 5).

In NKÜ Lider, shoot length remained unchanged under PEG-induced osmotic stress compared with the well-watered control but decreased by 10% under water scarcity. However, SNP, EBL, and SNP + EBL treatments increased shoot length by 13%, 3%, and 9%, respectively, under water scarcity (Figure 2a). In Kenanbey, shoot length decreased by 3% under PEG-induced osmotic stress and by 8% under water scarcity compared with the well-watered control. Under PEG-induced osmotic stress, EBL and SNP + EBL treatments decreased shoot length by 4% and 5%, respectively, whereas under water scarcity, SNP, EBL, and SNP + EBL treatments increased shoot length by 6%, 7%, and 4%, respectively, compared with the corresponding water-scarcity control (Figure 2b).

Root length was significantly reduced in Kenanbey by 20% under water scarcity compared with the well-watered control. Under water scarcity, SNP + EBL treatment significantly increased root length by 17% in both NKÜ Lider and Kenanbey varieties. In Kenanbey, EBL treatment also resulted in a significant 18% increase compared with the water-scarcity control (Figure 3a,b).

In the NKÜ Lider, PEG-induced osmotic stress and water scarcity reduced biomass by 17% and 32%, respectively, compared with the well-watered control. Within the well-watered, PEG, and water scarcity groups, neither SNP nor EBL treatment significantly affected biomass compared with the corresponding control, whereas the combined SNP + EBL treatment increased biomass by 8%, 55%, and 39%, respectively (Figure 4a). Similarly, in Kenanbey, PEG-induced osmotic stress and water scarcity reduced biomass by 10% and 27%, respectively, compared with the well-watered control. Under PEG-induced osmotic stress, SNP + EBL treatment increased biomass by 12% compared with the corresponding control. Under water scarcity, SNP, EBL, and SNP + EBL treatments increased biomass by 11%, 23%, and 18%, respectively, compared with the water-scarcity control (Figure 4b).

In the NKÜ Lider variety, PEG and water scarcity reduced DW by 21% and 36%, respectively, compared to the well-watered control. Combined treatments of SNP and EBL increased DW by 38% and 44%, respectively, compared to the untreated plants in the PEG and water scarcity groups (Figure 5a). In the Kenanbey variety, PEG and water scarcity reduced DW by 7% and 21%, respectively, compared to the well-watered control. However, in the water scarcity group, SNP, EBL, and SNP + EBL treatments increased dry weight by 14%, 32%, and 18%, respectively, compared to the untreated water scarcity group (Figure 5b). In both varieties, the biomass and DW reductions under water scarcity, the enhancements induced by SNP + EBL in the NKÜ Lider, and the improvements achieved by EBL and SNP + EBL in Kenanbey under water scarcity were statistically significant (*p* < 0.05), whereas differences among individual treatments in certain groups remained non-significant (*p* > 0.05) (Figure 4 and Figure 5).

### 2.2. Relative Water Content and SPAD Values

**Figure 6:**
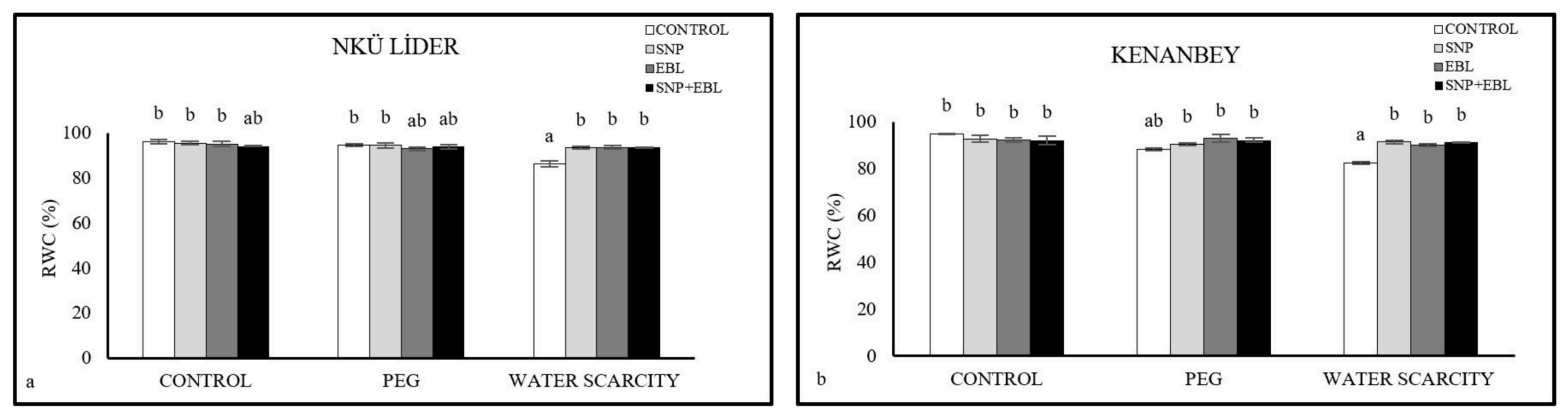
Effects of SNP and EBL treatments on RWC in NKÜ Lider (**a**) and Kenanbey (**b**) varieties under drought stress. Letters indicate statistically significant differences (*p* < 0.05).

Drought stress reduced RWC in the NKÜ Lider wheat variety. Relative to the well-watered control, RWC decreased by 2 and 10 percentage points (pp) under PEG-induced osmotic stress and water scarcity, respectively. Under PEG-induced osmotic stress, individual and combined treatments of SNP, EBL, and SNP + EBL did not significantly affect RWC compared to the untreated PEG control (*p* > 0.05). In contrast, under water scarcity, SNP, EBL, and SNP + EBL treatments each increased RWC by approximately 7 pp relative to the untreated water-scarcity control (*p* < 0.05), effectively restoring RWC levels closer to those of the well-watered control (Figure 6a). Similarly, drought stress led to significant reductions in RWC in the Kenanbey variety, decreasing by 7 and 12 pp under PEG-induced osmotic stress and water scarcity, respectively, compared to the well-watered control. Under PEG stress, treatments with SNP, EBL, or SNP + EBL resulted in no significant alterations in RWC relative to the untreated PEG control (*p* > 0.05). However, under water scarcity, RWC was significantly enhanced by 9, 8, and 9 pp following SNP, EBL, and SNP + EBL treatments, respectively, compared to the untreated water-scarcity control (*p* < 0.05), thereby mitigating the adverse effects of water scarcity on leaf water status (Figure 6b).

**Figure 7:**
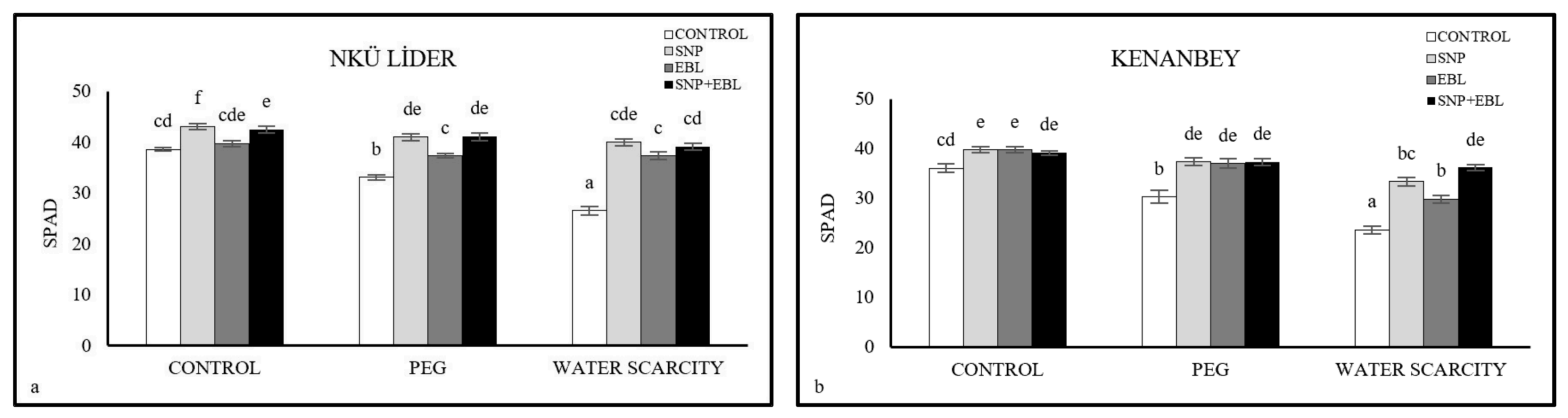
Effects of SNP and EBL treatments on SPAD in NKÜ Lider (**a**) and Kenanbey (**b**) varieties under drought stress. Letters indicate statistically significant differences (*p* < 0.05).

Drought stress significantly reduced SPAD values in both NKÜ Lider and Kenanbey wheat varieties. In NKÜ Lider, SPAD values decreased by 13% and 33% under PEG-induced osmotic stress and water scarcity, respectively, compared with the well-watered control. Under PEG-induced osmotic stress, SNP, EBL, and SNP + EBL treatments increased SPAD values by 24%, 13%, and 24%, respectively, relative to the untreated PEG control. Under water scarcity, the corresponding increases were 50%, 41%, and 47%, respectively (Figure 7a). In Kenanbey, SPAD values decreased by 16% and 35% under PEG-induced osmotic stress and water scarcity, respectively, compared with the well-watered control. Under PEG-induced osmotic stress, SNP, EBL, and SNP + EBL treatments increased SPAD values by approximately 23% in all treatment groups. Under water scarcity, these treatments increased SPAD values by 41%, 26%, and 53%, respectively, relative to the corresponding untreated stress controls (Figure 7b).

### 2.3. Oxidative Stress Markers

Drought stress increased H_2_O_2_ content, TBARS content, and EL, which are important indicators of oxidative stress, in both wheat varieties compared to the control.

**Figure 8:**
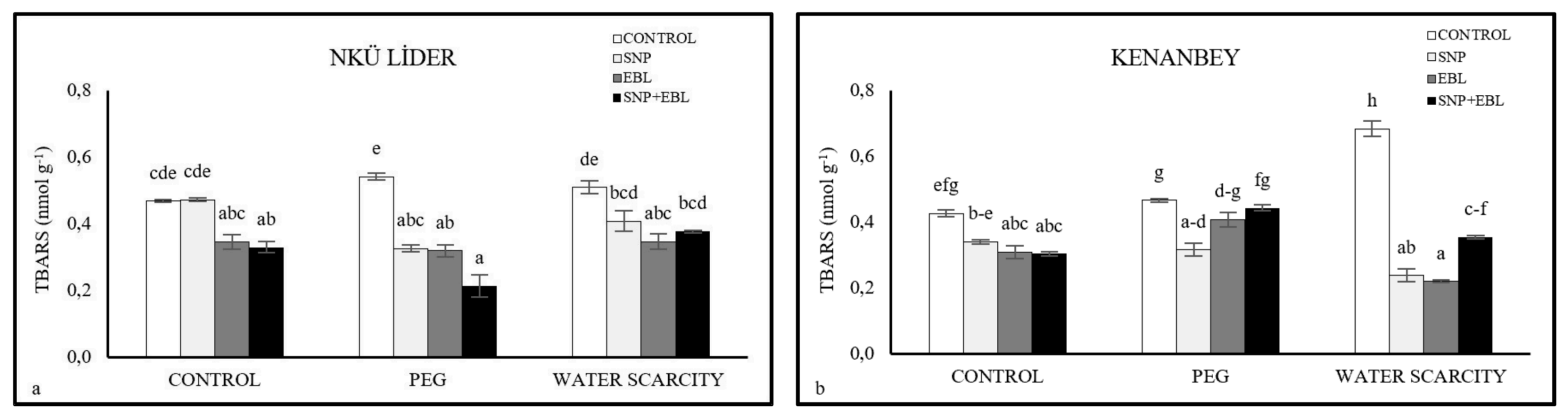
Effects of SNP and EBL treatments on lipid peroxidation levels in NKÜ Lider (**a**) and Kenanbey (**b**) varieties under drought stress. Letters indicate statistically significant differences (*p* < 0.05).

TBARS content, which is an indicator of lipid peroxidation and cell membrane damage, increased by 16% under PEG-induced osmotic stress and by 9% under water scarcity in the NKÜ Lider variety compared with the well-watered control. Under PEG-induced osmotic stress, SNP, EBL, and SNP + EBL treatments decreased TBARS content by 40%, 41%, and 61%, respectively, relative to the untreated PEG control. Under water scarcity, these treatments decreased TBARS content by 20%, 32%, and 16%, respectively, compared with the untreated water-scarcity control (Figure 8a).

In the Kenanbey variety, TBARS content increased by 9% under PEG-induced osmotic stress and by 60% under water scarcity compared with the well-watered control. Under PEG-induced osmotic stress, SNP, EBL, and SNP + EBL treatments decreased TBARS content by 32%, 12%, and 5%, respectively, relative to the untreated PEG control. Under water scarcity, these treatments decreased TBARS content by 65%, 68%, and 48%, respectively, compared with the untreated water-scarcity control (Figure 8b).

**Figure 9:**
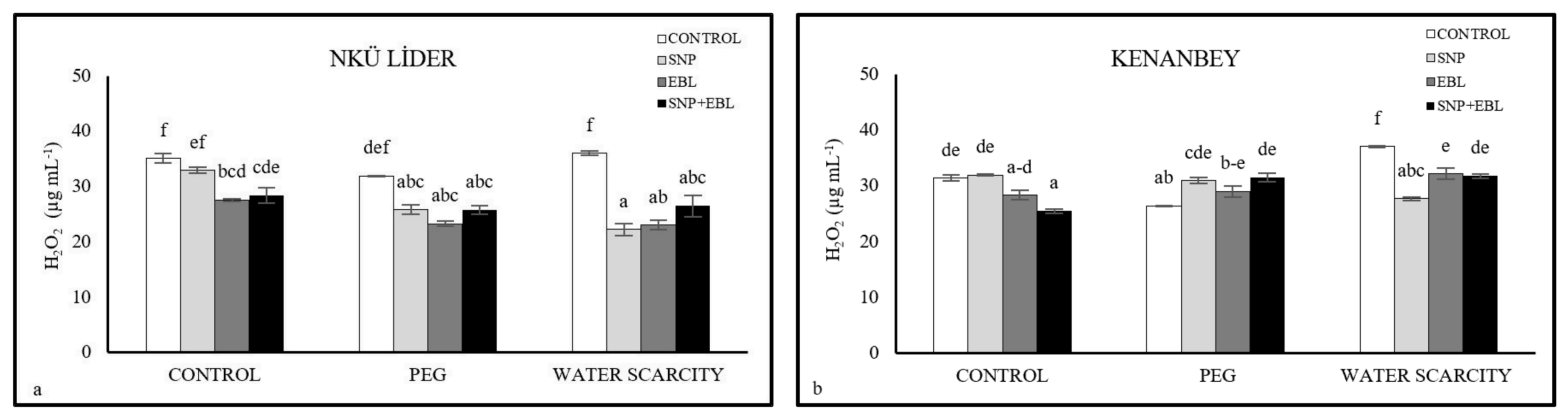
Effects of SNP and EBL treatments on H_2_O_2_ content in NKÜ Lider (**a**) and Kenanbey (**b**) varieties under drought stress. Letters indicate statistically significant differences (*p* < 0.05).

In the NKÜ Lider variety, PEG-induced osmotic stress and water scarcity did not alter H_2_O_2_ content compared with the well-watered control. Under PEG-induced osmotic stress, SNP, EBL, and SNP + EBL treatments decreased H_2_O_2_ content by 6%, 21%, and 19%, respectively, relative to the untreated PEG control. Under water scarcity, these treatments decreased H_2_O_2_ content by 38%, 36%, and 26%, respectively, compared with the untreated water-scarcity control (Figure 9a). In the Kenanbey variety, water scarcity increased H_2_O_2_ content by 18% compared with the well-watered control. Under water scarcity, SNP, EBL, and SNP + EBL treatments decreased H_2_O_2_ content by 25%, 13%, and 14%, respectively, relative to the untreated water-scarcity control (Figure 9b).

**Figure 10:**
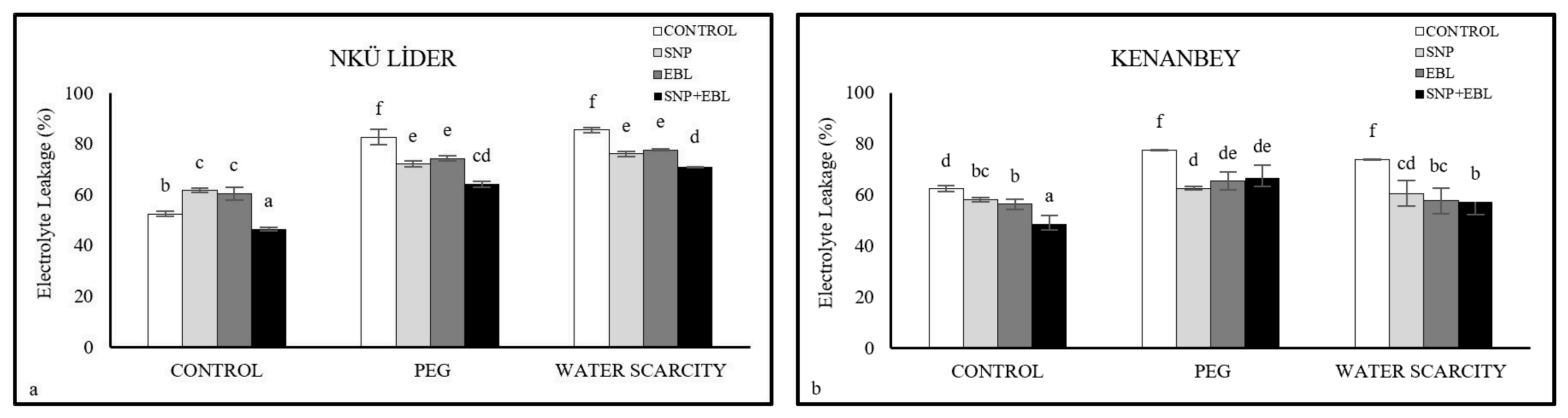
Effects of SNP and EBL treatments on EL in NKÜ Lider (**a**) and Kenanbey (**b**) varieties under drought stress. Letters indicate statistically significant differences (*p* < 0.05).

In the NKÜ Lider variety, EL increased by 57% and 63% under PEG-induced osmotic stress and water scarcity, respectively, compared with the well-watered control. Under PEG-induced osmotic stress, SNP, EBL, and SNP + EBL treatments decreased EL by 12%, 10%, and 22%, respectively, relative to the untreated PEG control. Under water scarcity, these treatments decreased EL by 11%, 9%, and 17%, respectively, compared with the untreated water-scarcity control (Figure 10a). In the Kenanbey variety, EL increased by 24% and 18% under PEG-induced osmotic stress and water scarcity, respectively, compared with the well-watered control. Under PEG-induced osmotic stress, SNP, EBL, and SNP + EBL treatments decreased EL by 19%, 15%, and 14%, respectively, relative to the untreated PEG control. Under water scarcity, these treatments decreased EL by 18%, 20%, and 21%, respectively, compared with the untreated water-scarcity control (Figure 10b).

### 2.4. Antioxidant Enzyme Activities

**Figure 11:**
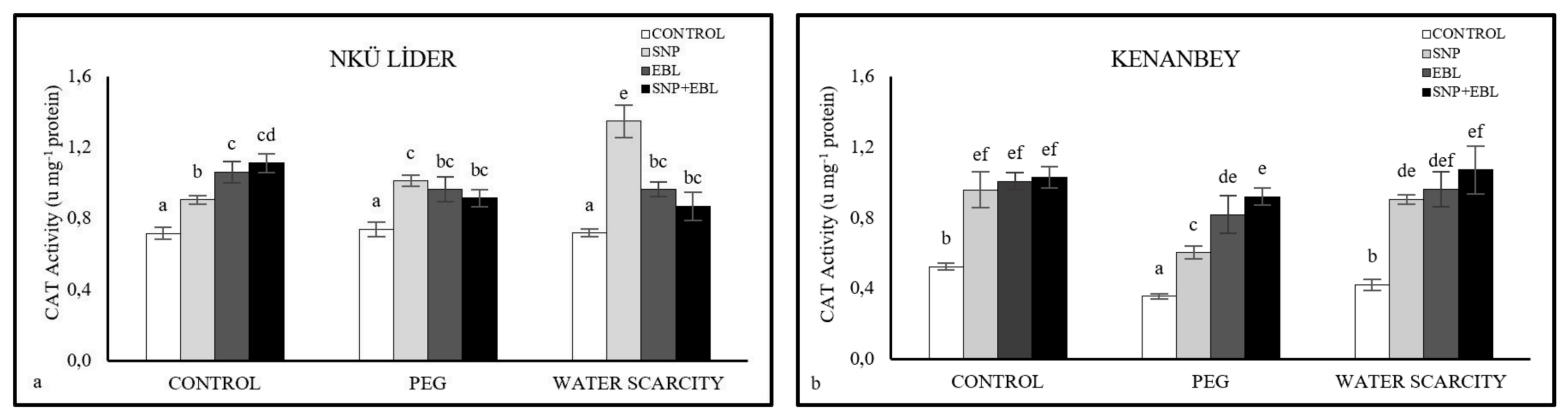
Effects of SNP and EBL treatments on CAT activity of NKÜ Lider (**a**) and Kenanbey (**b**) varieties under drought stress. Letters indicate statistically significant differences (*p* < 0.05).

In both varieties, individual and combined SNP and EBL treatments stimulated increases in antioxidant enzyme activities compared with the respective drought-stress treatments. CAT activity increased by 26%, 48%, and 55% with SNP, EBL, and SNP + EBL treatments, respectively, in the NKÜ Lider compared with the control. PEG-induced osmotic stress and water scarcity did not significantly alter CAT activity. However, in the PEG group, SNP, EBL, and SNP + EBL treatments increased CAT activity by 37%, 30%, and 24%, respectively, relative to the untreated PEG control. Under water scarcity, these increases were 87%, 34%, and 21%, respectively, compared with the untreated water-scarcity control (Figure 11a).

In the Kenanbey variety, SNP, EBL, and SNP + EBL treatments increased CAT activity by 83%, 92%, and 97%, respectively, compared with the control. In the control treatment, PEG and water scarcity reduced CAT activity by 32% and 20%, respectively. Under PEG-induced osmotic stress, SNP, EBL, and SNP + EBL treatments increased CAT activity by 70%, 130%, and 159%, respectively, relative to the untreated PEG control. Under water scarcity, these increases were 115%, 128%, and 154%, respectively, compared with the untreated water-scarcity control (Figure 11b).

**Figure 12:**
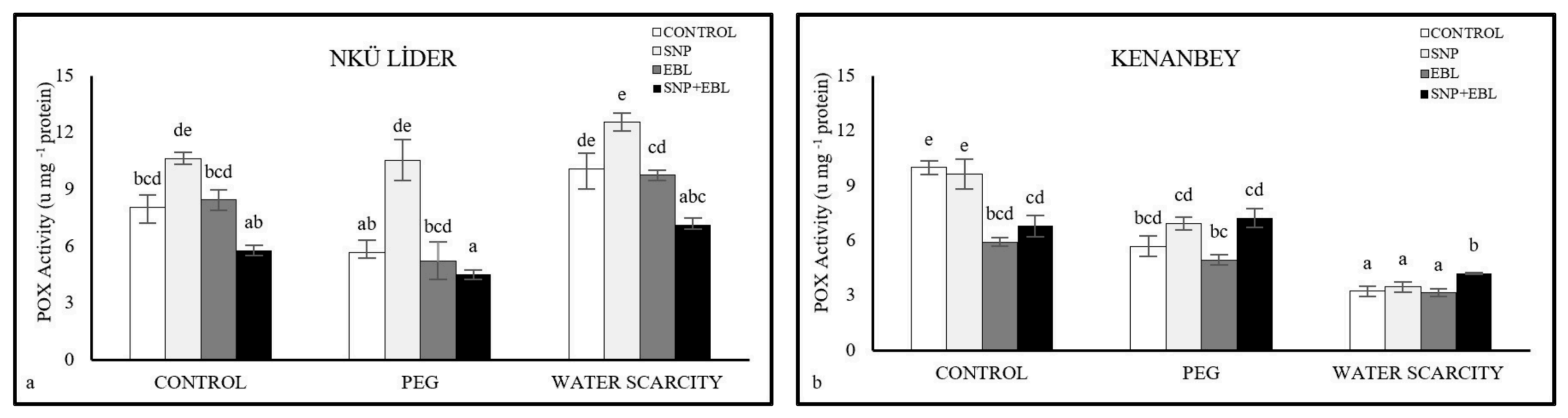
Effects of SNP and EBL treatments on POX activity of NKÜ Lider (**a**) and Kenanbey (**b**) varieties under drought stress. Letters indicate statistically significant differences (*p* < 0.05).

In the NKÜ Lider variety, POX activity decreased by 30% under PEG-induced osmotic stress but increased by 25% under water scarcity compared with the control. Under PEG-induced osmotic stress, SNP treatment increased POX activity by 86%, whereas EBL and SNP + EBL treatments decreased the activity by 8% and 20%, respectively, relative to the untreated PEG control. Under water scarcity, SNP treatment increased POX activity by 25%, whereas SNP + EBL treatment decreased it by 29% compared with the untreated water-scarcity control (Figure 12a). In the Kenanbey variety, POX activity decreased by 43% under PEG-induced osmotic stress and by 68% under water scarcity compared with the control. Under PEG-induced osmotic stress, SNP and SNP + EBL treatments increased POX activity by 22% and 27%, respectively, relative to the untreated PEG control. Under water scarcity, only the combined SNP + EBL treatment increased POX activity by 30%, compared with the untreated water-scarcity control (Figure 12b).

**Figure 13:**
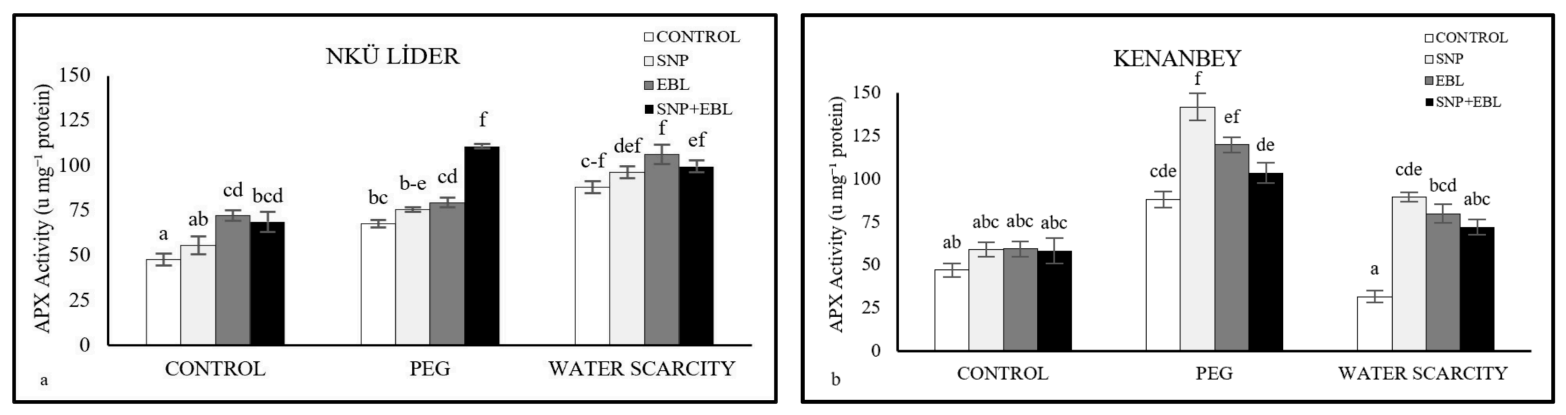
Effects of SNP and EBL treatments on APX activity of NKÜ Lider (**a**) and Kenanbey (**b**) varieties under drought stress. Letters indicate statistically significant differences (*p* < 0.05).

In the NKÜ Lider variety, APX activity increased by 42% under PEG-induced osmotic stress and by 84% under water scarcity compared with the control. SNP, EBL, and SNP + EBL treatments increased APX activity by 16%, 51%, and 41%, respectively, in the control group; by 12%, 17%, and 64%, respectively, under PEG-induced osmotic stress; and by 10%, 21%, and 13%, respectively, under water scarcity (Figure 13a). In the Kenanbey variety, all treatments increased APX activity by 25% in the control group. APX activity increased by 87% under PEG-induced osmotic stress but decreased by 33% under water scarcity compared with the control. Under PEG-induced osmotic stress, SNP, EBL, and SNP + EBL treatments increased APX activity by 61%, 36%, and 17%, respectively. Under water scarcity, these treatments increased APX activity by 184%, 153%, and 128%, respectively (Figure 13b).

**Figure 14:**
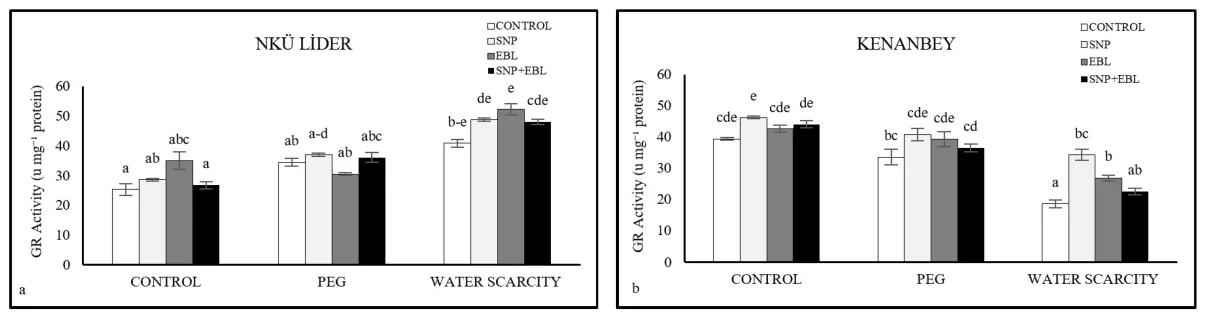
Effects of SNP and EBL treatments on GR activity of NKÜ Lider (**a**) and Kenanbey (**b**) varieties under drought stress. Letters indicate statistically significant differences (*p* < 0.05).

In the NKÜ Lider variety, GR activity increased by 36% under PEG-induced osmotic stress and by 61% under water scarcity compared with the well-watered control. Under PEG-induced osmotic stress, EBL decreased GR activity by 11%, whereas SNP and SNP + EBL increased it by 7% and 5%, respectively. Under water scarcity, SNP, EBL, and SNP + EBL increased GR activity by 20%, 28%, and 18%, respectively, compared with the corresponding water-scarcity control (Figure 14a). In Kenanbey, GR activity decreased by 15% under PEG-induced osmotic stress and by 53% under water scarcity compared with the well-watered control. Under PEG-induced osmotic stress, SNP, EBL, and SNP + EBL increased GR activity by 22%, 17%, and 9%, respectively, whereas under water scarcity, the increases were 84%, 44%, and 21%, respectively, compared with the corresponding stress controls (Figure 14b).

### 2.5. Isoenzyme Profiles

**Figure 15:**
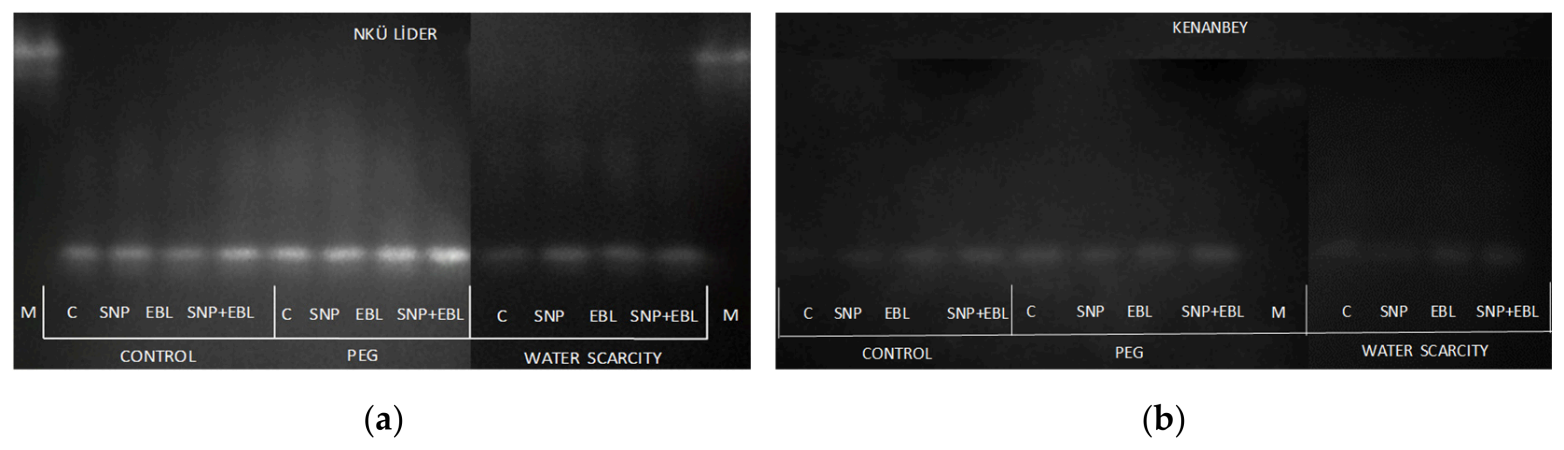
Superoxide dismutase (SOD) isoenzyme profiles in wheat (*Triticum aestivum* L.) under drought stress: (**a**) NKÜ Lider; (**b**) Kenanbey. Plants were subjected to control or drought (PEG and water scarcity) conditions and treated with C (control), SNP (sodium nitroprusside), EBL (24-epibrassinolide), or their combination, SNP + EBL. (M: Marker).

**Figure 16:**
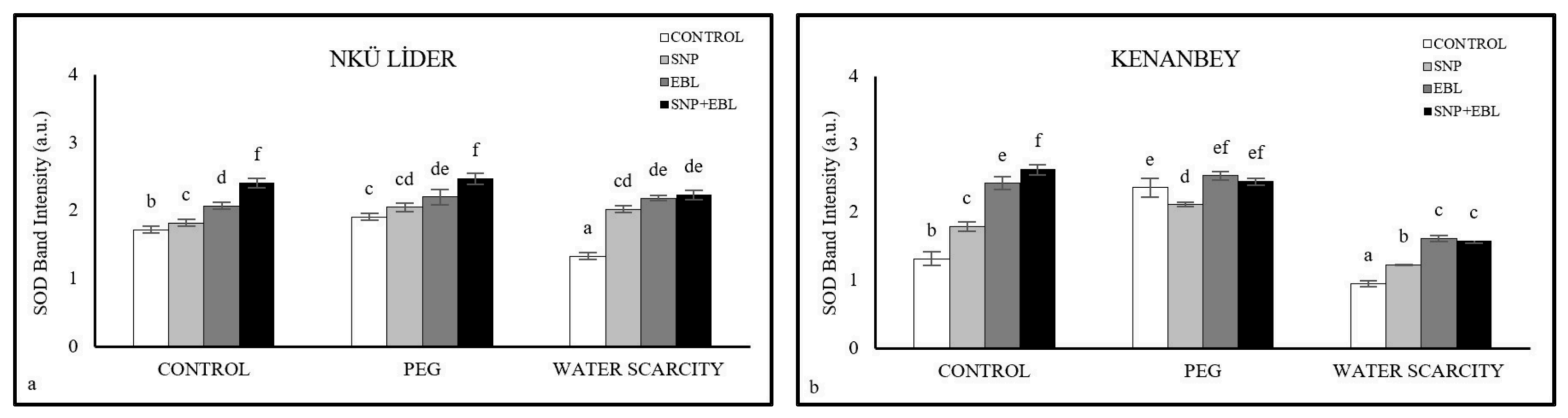
Effects of SNP and EBL treatments on SOD isoenzyme band intensity in the NKÜ Lider (**a**) and Kenanbey (**b**) wheat varieties under PEG-induced osmotic stress and water scarcity. Bars represent means ± SE (*n* = 3). Different letters indicate statistically significant differences (*p* < 0.05), whereas bars sharing at least one letter are not significantly different. Band intensity is expressed in arbitrary densitometric units.

In the NKÜ Lider variety, SOD isoenzyme intensity in the control treatment increased by 11% under PEG-induced osmotic stress but decreased by 23% under water scarcity, relative to the well-watered control. Under well-watered conditions, SNP, EBL, and SNP + EBL increased SOD isoenzyme intensity by 6%, 21%, and 40%, respectively, relative to the control treatment within the same water-status condition. Under PEG-induced osmotic stress, SNP, EBL, and SNP + EBL increased intensity by 7%, 15%, and 29%, respectively, compared with the PEG control. Under water scarcity, these treatments increased SOD isoenzyme intensity by 52%, 64%, and 68%, respectively, compared with the water-scarcity control (Figure 15a and Figure 16a). In the Kenanbey variety, SOD isoenzyme intensity in the control treatment increased by 79% under PEG-induced osmotic stress but decreased by 28% under water scarcity, relative to the well-watered control. Under well-watered conditions, SNP, EBL, and SNP + EBL increased intensity by 36%, 84%, and 100%, respectively, relative to the well-watered control treatment. Under PEG-induced osmotic stress, SNP decreased intensity by 10%, whereas EBL and SNP + EBL increased it by 8% and 4%, respectively, relative to the PEG control. Under water scarcity, SNP, EBL, and SNP + EBL increased SOD isoenzyme intensity by 28%, 69%, and 66%, respectively, relative to the water-scarcity control (Figure 15b and Figure 16b).

**Figure 17:**
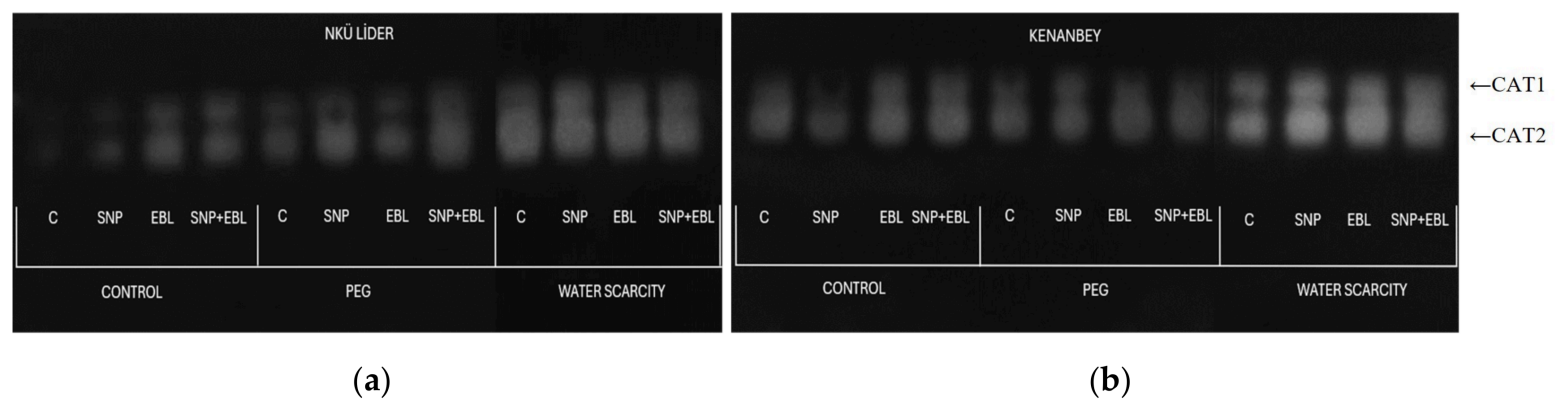
Catalase (CAT1 and CAT2) isoenzyme profiles in wheat (*Triticum aestivum* L.) under drought stress: (**a**) NKÜ Lider; (**b**) Kenanbey. Plants were subjected to control or drought (PEG and water scarcity) conditions and treated with C (control), SNP (sodium nitroprusside), EBL (24-epibrassinolide), or their combination SNP + EBL.

**Figure 18:**
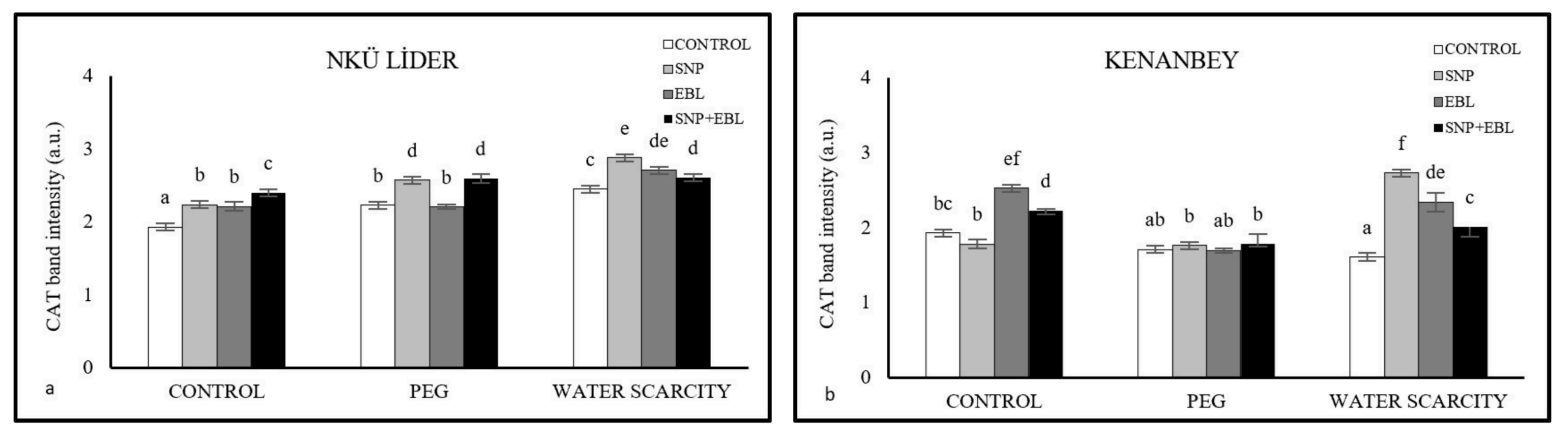
Effects of SNP and EBL treatments on CAT isoenzyme band intensity in the NKÜ Lider (**a**) and Kenanbey (**b**) wheat varieties under PEG-induced osmotic stress and water scarcity. Bars represent means ± SE (*n* = 3). Different letters indicate statistically significant differences (*p* < 0.05), whereas bars sharing at least one letter are not significantly different. Band intensity is expressed in arbitrary densitometric units.

CAT isoenzyme profiles (CAT1 and CAT2) varied between wheat varieties and drought treatments (Figure 17 and Figure 18). In the NKÜ Lider variety, CAT isoenzyme band intensity increased by 16% under PEG-induced osmotic stress and by 27% under water scarcity relative to the well-watered control. Under PEG-induced osmotic stress, SNP and SNP + EBL treatments increased band intensity by 15% and 17%, respectively, compared with the untreated PEG control. Under water scarcity, SNP, EBL, and SNP + EBL treatments increased band intensity by 18%, 10%, and 6%, respectively, relative to the untreated water-scarcity control (Figure 17a and Figure 18a).

In the Kenanbey variety, CAT isoenzyme band intensity decreased by 11% under PEG-induced osmotic stress and by 17% under water scarcity relative to the well-watered control. Under water scarcity, SNP, EBL, and SNP + EBL treatments increased band intensity by 70%, 45%, and 25%, respectively, compared with the untreated water-scarcity control (Figure 17b and Figure 18b).

### 2.6. Histochemical Analyses

**Figure 19:**
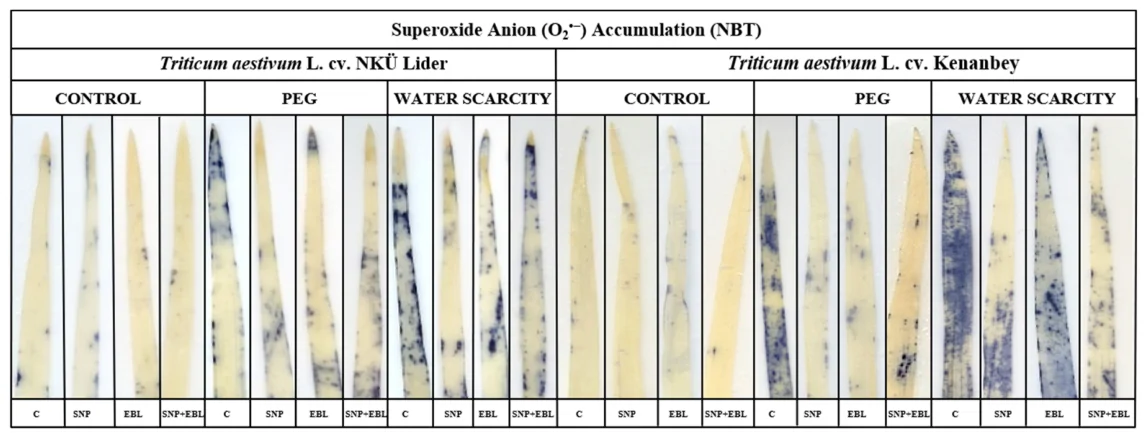
Histochemical detection of superoxide anion (O_2_^•−^) accumulation in leaves of two wheat varieties (*Triticum aestivum* L., NKÜ Lider and Kenanbey) under drought stress. O_2_^•−^ localization was visualized using nitroblue tetrazolium (NBT) staining. Plants were subjected to control and drought stress conditions (PEG and water scarcity) and treated with C (control), SNP (sodium nitroprusside), EBL (24-epibrassinolide), or their combination (SNP + EBL). The blue to dark-blue formazan deposits indicate the sites of (O_2_^•−^) accumulation.

The detection of O_2_^•−^ accumulation through formazan formation following NBT reflects the effect of stress. O_2_^•−^ accumulation in the leaves increased under PEG-induced osmotic stress and water scarcity compared with the well-watered control. This increase was greater in Kenanbey than in NKÜ Lider. In both varieties, the combined SNP + EBL treatment reduced staining intensity, indicating a reduction in O_2_^•−^ accumulation. NBT staining intensity in the SNP, EBL, and SNP + EBL treatment groups was comparable to that of the well-watered control, whereas stronger staining was observed under PEG-induced osmotic stress and water scarcity (Figure 19).

**Figure 20:**
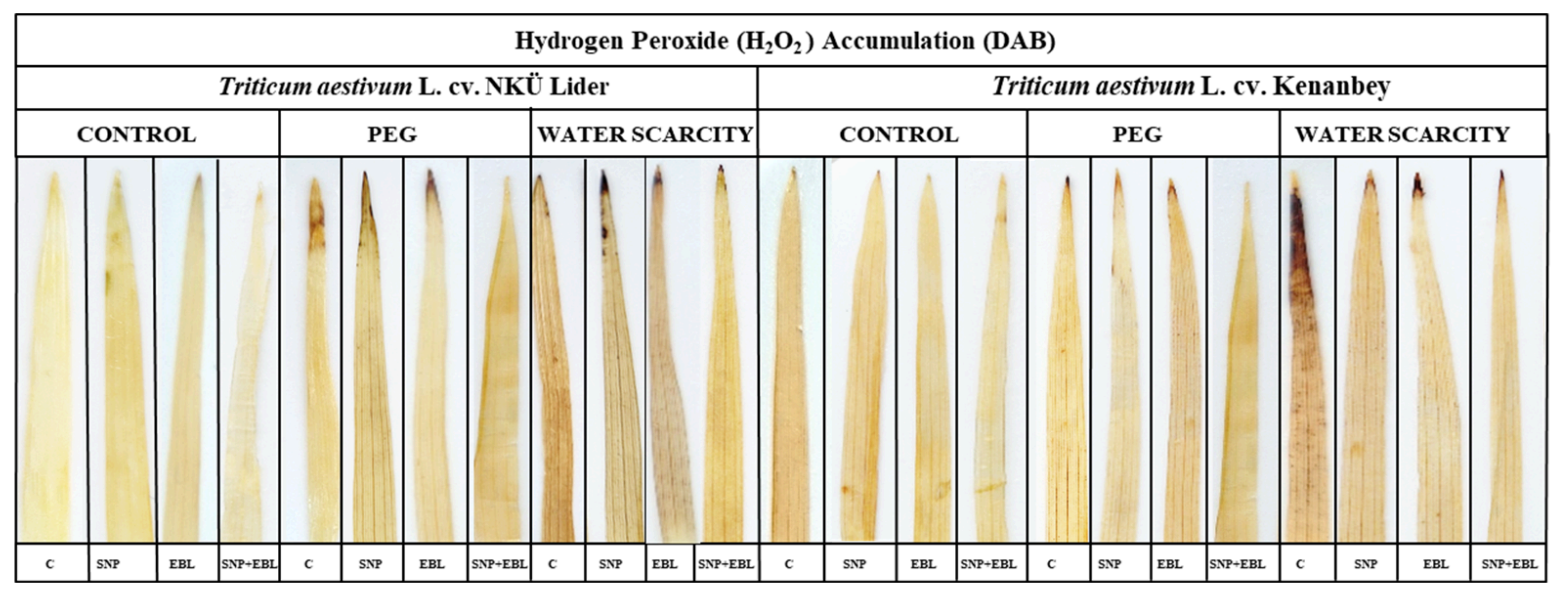
Histochemical detection of hydrogen peroxide (H_2_O_2_) accumulation in leaves of two wheat varieties (*Triticum aestivum* L. cv. NKÜ Lider and Kenanbey) under drought stress. H_2_O_2_ localization was visualized using 3,3′-diaminobenzidine (DAB) staining. Plants were subjected to control and drought stress conditions (PEG and water scarcity) and treated with C (control), SNP (sodium nitroprusside), EBL (24-epibrassinolide), or their combination (SNP + EBL). The dark brown deposits indicate the sites of H_2_O_2_ accumulation, particularly localized at the leaf tips under water scarcity conditions.

Histochemical staining for H_2_O_2_ detection showed high accumulation in the leaves of both varieties in the water-scarcity group compared with the well-watered control, and this accumulation was localized at the leaf tips. In the SNP + EBL group, despite PEG-induced osmotic stress and water scarcity, the H_2_O_2_ content of both varieties was found to be at a similar level to the well-watered control group (Figure 20).

**Figure 21:**
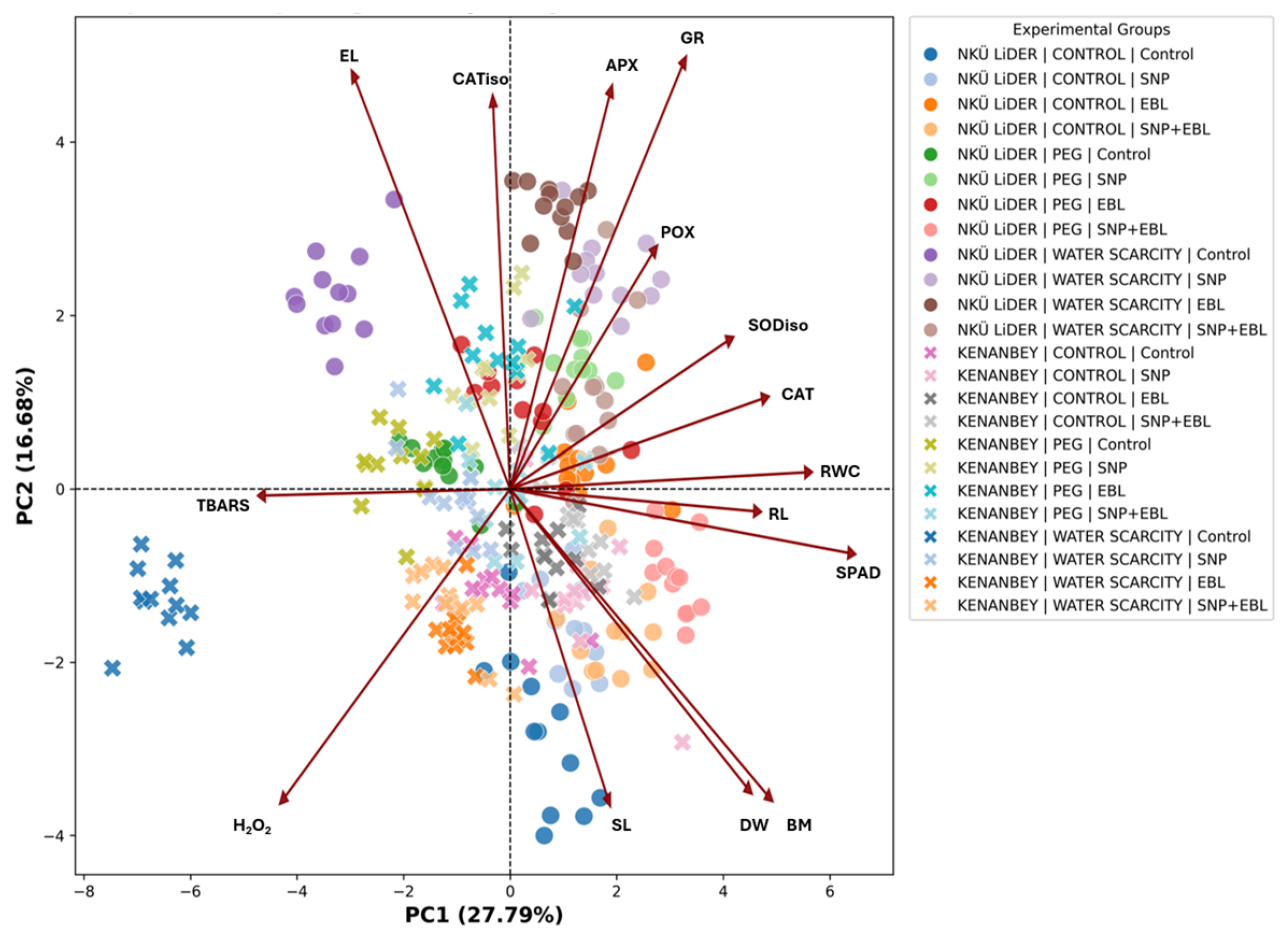
PCA biplot of physiological and biochemical parameters in two wheat varieties (*Triticum aestivum* L. cv. NKÜ Lider and Kenanbey) under drought stress. The biplot illustrates the distribution of experimental groups and the loading vectors of the analyzed traits along the first two principal components (PC1: 27.79% and PC2: 16.68%). Plants were subjected to control and drought stress conditions (PEG and Water Scarcity) and treated with Control (untreated), SNP (sodium nitroprusside), EBL (24-epibrassinolide), or their combination (SNP + EBL). Circles and crosses represent the NKÜ Lider and Kenanbey varieties, respectively, while colors denote specific treatment combinations (APX, ascorbate peroxidase; BM, biomass; CAT, catalase; CATiso, catalase isoenzyme; DW, dry weight; EL, electrolyte leakage; GR, glutathione reductase; H_2_O_2_, hydrogen peroxide; POX, peroxidase; RL, root length; RWC, relative water content; SL, shoot length; SODiso, superoxide dismutase isoenzyme; SPAD; TBARS, lipid peroxidation).

To comprehensively evaluate the multivariate responses of the two wheat varieties with contrasting drought tolerances, a Principal Component Analysis (PCA) was performed (Figure 21). The first two principal components accounted for 44.47% of the total cumulative variance, with PC1 and PC2 explaining 27.79% and 16.68%, respectively. The biplot demonstrated clear discrimination between the drought-tolerant variety NKÜ Lider and the drought-sensitive variety Kenanbey, alongside distinct separation between control and stress treatments. Growth parameters such as RWC, SPAD value, RL, SL, DW, and BM clustered positively along the positive axis of PC1. Conversely, oxidative stress and membrane damage indicators, including H_2_O_2_, TBARS, and EL, loaded heavily on the negative axis of PC1. The drought-sensitive variety Kenanbey under water-scarcity conditions formed an isolated cluster at the extreme lower-left quadrant, characterized by low RWC and SPAD values coupled with high H_2_O_2_ and TBARS levels. In contrast, NKÜ Lider, particularly under PEG and water scarcity combined with SNP, EBL, and SNP + EBL, clustered closely with enzymatic antioxidant vectors such as CAT, SODiso, CATiso, POX, APX, and GR in the upper regions. Treatments of SNP, EBL, and SNP+ EBL induced a notable shift in the sensitive variety Kenanbey away from the stress-vulnerable zone toward the central coordinate plane.

**Figure 22:**
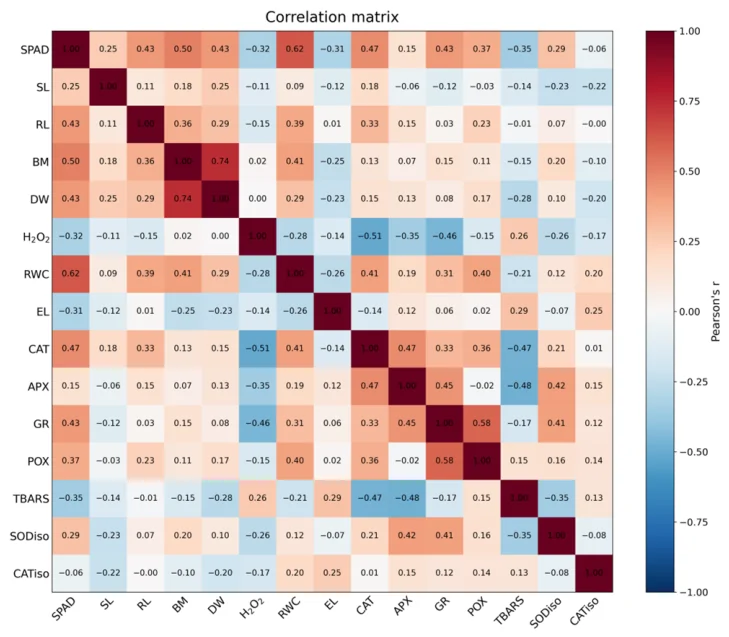
Pearson’s correlation matrix of physiological and biochemical parameters in two wheat varieties (*Triticum aestivum* L., cv. NKÜ Lider and Kenanbey) under drought stress and exogenous treatments. The heatmap visualizes the correlation coefficients (*r*) among the evaluated traits. Positive correlations are represented by red color gradients, whereas negative correlations are depicted by blue color gradients. The intensity of the color and the numerical values within each cell indicate the strength of the linear relationship between variables, ranging from −1.00 (strong negative correlation) to 1.00 (strong positive correlation) (APX, ascorbate peroxidase; BM, biomass; CAT, catalase; CATiso, catalase isoenzyme; DW, dry weight; EL, electrolyte leakage; GR, glutathione reductase; H_2_O_2_, hydrogen peroxide; POX, peroxidase; RL, root length; RWC, relative water content; SL, shoot length; SODiso, superoxide dismutase isoenzyme; SPAD; TBARS, lipid peroxidation).

To further elucidate the interrelationships among growth and antioxidant parameters across all treatments and varieties, a Pearson correlation analysis was conducted (Figure 22). Growth parameters exhibited positive interrelationships, with strong positive correlations observed between BM and DW (*r* = 0.74), SPAD and RWC (*r* = 0.62), and GR and POX (*r* = 0.58). Specifically, H_2_O_2_ showed an inverse association with CAT (*r* = −0.51), while TBARS correlated negatively with APX (*r* = −0.48) and CAT (*r* = −0.47).

## 3. Discussion

In this study, the effects of NO and BR, applied individually and together, were investigated in two wheat varieties with different drought tolerance (drought-tolerant NKÜ Lider and drought-sensitive Kenanbey). Both varieties were negatively affected by PEG-induced osmotic stress and water scarcity, and SNP and EBL alleviated many of these effects. However, responses varied among traits, varieties and stress regimes, and the combined SNP + EBL treatment was not superior to the individual treatments for every parameter.

Tolerance was not expressed uniformly across traits. Under water scarcity, NKÜ Lider exhibited larger proportional reductions in biomass (32%) and dry weight (DW; 36%) relative to the well-watered control than Kenanbey (27% and 21%, respectively). In contrast, RL suppression (20% relative to the well-watered control) and lipid peroxidation (TBARS increase of 60%) were more prominent in Kenanbey. Exogenous treatments partially counteracted these growth reductions, although responses were variety- and treatment-dependent. In NKÜ Lider, combined SNP + EBL treatment significantly enhanced DW by 38% under PEG and 44% under water scarcity compared with the corresponding untreated stress controls. In Kenanbey, under water scarcity, the greatest significant increase in DW was achieved with individual EBL treatment (32%), followed by SNP + EBL (18%) and SNP (14%) relative to the untreated water-scarcity control. Relative increases in SL, biomass and DW under water scarcity were larger in the NKÜ Lider, whereas RL, RWC and SPAD recovered similarly in both varieties.

SPAD values decreased by 13% and 33% in the NKÜ Lider and by 16% and 35% in the Kenanbey under PEG and water scarcity, respectively. Maintaining chlorophyll under stress is associated with drought tolerance through antioxidant defense, osmotic regulation and membrane integrity [24], whereas insufficient soil moisture and limited root activity reduce nutrient uptake [25]. Chlorophyll content declines to varying degrees in different wheat varieties according to their drought tolerance [26], and NO and BR have been reported to increase chlorophyll content in wheat [1,6,27]. Under PEG-induced osmotic stress, all three treatments were similarly effective within each variety. Under water scarcity, SNP + EBL produced the largest increase in the Kenanbey (53% over the untreated water-scarcity control), whereas in NKÜ Lider the increases elicited by SNP alone (50%) and SNP + EBL (47%) were statistically indistinguishable based on post hoc Tukey tests. RWC declines as drought progresses [28], and SNP helps maintain RWC [29,30,31,32]. Here, while treatments had no significant effect on RWC under moderate PEG stress, all treatments significantly restored leaf hydration under water scarcity, increasing RWC by approximately 7 percentage points (pp) in NKÜ Lider and 8–9 pp in the Kenanbey relative to their respective untreated water-scarcity controls, which may have contributed to the better growth of the Kenanbey.

NO is known to interact with other hormones and signaling molecules to activate antioxidant defense under drought [16,33,34,35], and the SNP concentration used here (200 µM) lies within the range applied in earlier wheat studies (100–200 µM) [32,34,36].

Drought-induced oxidative stress increases lipid peroxidation and EL [32,37,38]. Compared with the well-watered control, water scarcity induced substantial oxidative injury, evidenced by sharp increases in EL and TBARS, particularly in the Kenanbey (60% TBARS increase). Relative to the untreated stress controls, SNP and EBL treatments significantly attenuated lipid peroxidation and EL. However, the protective superiority of combined treatment was trait- and stress-specific: in NKÜ Lider, SNP + EBL conferred the greatest significant reduction in EL under both PEG (22%) and water scarcity (17%). Conversely, in Kenanbey under PEG stress, individual SNP reduced EL more effectively than SNP + EBL (19% vs. 14%), while under water scarcity, the reductions achieved by individual EBL (20%) and SNP + EBL (21%) did not differ significantly. Spectrophotometric H_2_O_2_ did not change in the NKÜ Lider and increased only in the Kenanbey under water scarcity (18%), whereas DAB staining showed H_2_O_2_ accumulation at the leaf tips of both varieties under water scarcity; localized increases may be diluted in whole-leaf extracts. NBT staining was weaker in treated leaves, suggesting reduced O_2_ ^•−^ accumulation, but this qualitative evidence was interpreted only as supportive [39]. The reductions in EL and TBARS coincided with enhanced antioxidant responses, which may have contributed to membrane protection, although this association does not prove causality.

SOD converts O_2_^•−^ to H_2_O_2_, which is subsequently detoxified by CAT, APX, and POX [10,11,40]. In the present study, drought-induced alterations in antioxidant enzyme activities and isoenzyme profiles exhibited clear variety- and stress-dependent patterns. While PEG-induced osmotic stress stimulated SOD isoenzyme band intensity, progressive water scarcity suppressed it in both varieties, though exogenous SNP and EBL treatments effectively counteracted this decline. Similarly, GR activity increased in NKÜ Lider under both stress regimes but was severely inhibited in Kenanbey, where exogenous SNP markedly restored its activity. These contrasting responses may indicate differences between the two wheat varieties in their ability to regulate the ascorbate–glutathione cycle under oxidative stress [41]. Drought-induced oxidative damage in wheat is primarily driven by excessive ROS accumulation, which triggers membrane lipid peroxidation and functional decay [6,42]. Under water scarcity, Kenanbey exhibited pronounced increments in both H_2_O_2_ accumulation and TBARS levels, whereas NKÜ Lider sustained lower baseline oxidative stress and limited lipid peroxidation (Figure 8 and Figure 9). This physiological divergence aligns with earlier findings indicating that drought-tolerant wheat varieties maintain superior redox buffering capacity and accumulate lower MDA and H_2_O_2_ than sensitive ones [28,43,44,45,46,47,48,49,50]. Exogenous SNP and EBL treatments effectively suppressed TBARS and H_2_O_2_ accumulation across both water regimes, which is consistent with reported NO- and BR-mediated protection of cellular membranes against dehydration [16,36,51,52,53,54]. Mechanistically, NO acts as a free radical scavenger and coordinates with BRs to upregulate core scavenging enzymes (SOD, CAT, APX, and GR), thereby protecting the ascorbate–glutathione cycle, safeguarding membrane integrity (reduced EL), and alleviating cellular damage under severe water scarcity [21,36,41,55]. The complementary effects of SNP and EBL may involve crosstalk between NO- and BR-associated signaling [13,56,57,58]. ROS are themselves multidimensional regulators of plant adaptation to abiotic stress, so treatment-induced changes in ROS metabolism may feedback on NO and hormone signaling [55]. In pepper under water stress, BR-induced NO production contributed to BR-induced antioxidant defense [21], and recent evidence shows that NO interacts with BR signaling in an organ-specific manner [22]. It can therefore be hypothesized that (i) EBL stimulates endogenous NO production, and NO acts downstream of BR to modulate the expression or activity of antioxidant enzymes, and (ii) NO and BR cooperate in regulating stomatal movement, root growth and osmolyte accumulation, thereby helping to maintain leaf water status. These hypotheses are consistent with the enhanced antioxidant responses, the partial restoration of RWC and the reductions in H_2_O_2_, TBARS and EL observed here under water scarcity. PCA revealed multivariate differences in the physiological and biochemical responses of NKÜ Lider and Kenanbey to drought stress. Pearson correlation analysis further showed positive associations among growth parameters and inverse associations between oxidative stress indicators and antioxidant enzyme activities. Differences between the NKÜ Lider and the Kenanbey may largely stem from the seedling stage examined and the inherent tolerance thresholds of the two varieties. Under water scarcity, biomass, DW, RL, TBARS and SOD isoenzyme band intensity were affected in both varieties. In the NKÜ Lider, the combined treatment was particularly effective in improving RL, biomass, DW and SOD isoenzyme profile while reducing EL; in the Kenanbey, it markedly enhanced biomass, SPAD values, CAT and POX activities and SOD isoenzyme intensity, along with attenuating EL.

From a practical perspective, the most promising effects for further testing were the partial restoration of RWC and SPAD values under water scarcity, the reduction in membrane damage (EL and TBARS) and the modulation of antioxidant defenses, whereas effects on growth traits varied with variety and stress regime. This experiment tested a single dose of each compound, applied once to 21-day-old seedlings, with a 7-day stress period under controlled conditions; grain yield, reproductive-stage drought, repeated applications, field performance and substrate water status were not evaluated, so an optimal dose or regime cannot be inferred. Dose–response, application-timing and field studies [59], combined with molecular analyses and digital modeling, are needed to establish agronomic applicability [60]. Under the conditions of this experiment, foliar application of 200 µM SNP + 0.05 mg L^−1^ EBL alleviated drought-induced oxidative damage in seedlings of both wheat varieties, with a more pronounced improvement in the drought-sensitive variety; however, its field application potential still needs to be verified.

## 4. Materials and Methods

### 4.1. Plant Materials and Growth Conditions

In this study, two bread wheat (*Triticum aestivum* L.) varieties with contrasting drought tolerance, NKÜ Lider and Kenanbey, were used [42]. The seeds were surface-sterilized in a 5% sodium hypochlorite (NaOCl) solution for 5 min and then rinsed three times with distilled water. The sterilized seeds were first germinated in sterile Petri dishes and subsequently transferred to 3 L pots (18 × 20 cm) containing perlite, with 20 seedlings per pot. In the growth chamber, the plants were maintained at 25 ± 2 °C under a 16 h light/8 h dark photoperiod, with a relative humidity of 70–75% and a light intensity of 300 µmol m^−2^ s^−1^. The plants were watered with 100% Hoagland nutrient solution [61]. The experimental design given in Table 1 was used for each variety. Each variety × treatment × water-status combination consisted of three independent pots. Three plants were sampled from each pot. The pots served as biological experimental units, with three biological replicates per combination (*n* = 3).

### 4.2. Experimental Treatments

Sodium nitroprusside (SNP) was used as a nitric oxide (NO) donor at a concentration of 200 µM [16]. SNP was dissolved in distilled water and sprayed onto the leaves of 21-day-old seedlings at a volume of 25 mL per pot. For brassinosteroid treatment, 24-epibrassinolide (EBL) was initially dissolved in 1 mL ethanol and subsequently diluted with distilled water to prepare the stock solution. EBL was applied exogenously as a foliar spray at a concentration of 0.05 mg L^−1^, using 25 mL per pot [62].

Two wheat varieties differing in drought tolerance, namely the drought-tolerant NKÜ Lider and the drought-sensitive Kenanbey, were subjected to treatments consisting of SNP (200 µM), EBL (0.05 mg L^−1^), and their combination (200 µM SNP + 0.05 mgL^−1^ EBL) applied exogenously to the leaves.

Drought stress was evaluated under three distinct water regimes: well-watered control, moderate osmotic stress induced by adding 15% (*w*/*v*) polyethylene glycol (PEG)-6000 to the Hoagland nutrient solution, and progressive water scarcity imposed by withholding the nutrient solution [63]. The osmotic potential of the 15% (*w*/*v*) PEG-6000 solution (−0.30 MPa) was calculated according to Michel and Kaufmann [23]. Because substrate water status and water potential were not directly measured, PEG-induced osmotic stress and water scarcity were treated as distinct stress regimes.

Plants were harvested on the 7th day of stress for physiological and biochemical analyses. Shoot and root length, biomass, dry weight, and relative water content (RWC) were evaluated in both varieties. Biochemical analyses included antioxidant enzyme activities (POX, APX, GR, and CAT), isoenzyme profiles (SOD and CAT), SPAD value, lipid peroxidation (TBARS), hydrogen peroxide (H_2_O_2_) content, and electrolyte leakage (EL). Furthermore, the histochemical accumulation of H_2_O_2_ and O_2_^•−^ was determined in leaf tissues.

### 4.3. Physiological Analyses

The shoot length, root length, biomass, dry weight, SPAD and relative water content were measured.

#### 4.3.1. Shoot Length (SL) and Root Length (RL)

In all experimental groups, the shoot length (measured from the root collar to the shoot apex) and root length (measured from the root collar to the root tip) of the seedlings were determined in centimeters using a ruler.

#### 4.3.2. Relative Water Content (RWC)

Relative water content (RWC) was determined using the method described by [64]. The youngest fully expanded leaves were selected for RWC determination. Fresh leaf samples were weighed immediately after harvesting to determine their fresh weight (FW). The leaves were then floated on distilled water in Petri dishes between layers of filter paper for 4 h to obtain their turgid weight (TW). Subsequently, the samples were dried in an oven at 70 °C for 24 h, and their dry weight (DW) was recorded. RWC was calculated according to the following Equation (1):

%RWC = ((FW − DW) / (TW − DW)) × 100(1)

#### 4.3.3. Biomass (BM)

Biomass was determined by using three seedlings from each of three independent pots per treatment (nine seedlings in total, *n* = 9). Root and shoot tissues were weighed together. Before weighing, roots were washed with water to remove adhering growth medium, and excess surface water was removed using Whatman filter paper. Fresh biomass was expressed as g plant^−1^.

#### 4.3.4. Dry Weight (DW)

To determine dry weight, the same seedlings used for BM determination, including both root and shoot tissues, were dried in an oven at 70 °C for 48 h and weighed using a precision balance. Dry weight was expressed as g plant^−1^, and the mean values were calculated.

#### 4.3.5. SPAD Value

The SPAD value was determined using a Minolta SPAD-502 chlorophyll meter (Konica Minolta, Inc., Tokyo, Japan) on fully expanded wheat leaves of the plants [65]. Three plants were selected from each of three independent pots per treatment (*n* = 9). Three readings were taken at different positions along each leaf, and the mean of these readings was used for subsequent analyses.

### 4.4. Biochemical Analyses

The hydrogen peroxide, lipid peroxidation, electrolyte leakage, protein content, and antioxidant enzyme activities were determined using standard biochemical protocols.

#### 4.4.1. Lipid Peroxidation Content (TBARS)

Leaf tissues (0.1 g) were homogenized with 0.1% (*w*/*v*) trichloroacetic acid (TCA) solution. The homogenate was centrifuged, and the supernatant was transferred into new tubes. A reaction mixture containing TCA and thiobarbituric acid (TBA) was added to the supernatant, and the mixture was incubated in a boiling water bath at 95 °C for 15 min. Subsequently, the reactions were stopped by placing the tubes in an ice bath, followed by centrifugation at 10,000× *g* for 5 min. The absorbance of the supernatant was recorded at 532 nm and 600 nm using a spectrophotometer, and the malondialdehyde (MDA) content was calculated accordingly [66].

#### 4.4.2. Hydrogen Peroxide Content (H_2_O_2_)

Plant samples (0.1 g) were homogenized with 1 mL of 100 mM sodium phosphate (Na-P) buffer (pH 6.8) and centrifuged. Then, 0.5 mL of the supernatant was mixed with 2.5 mL of the peroxide reagent. Following incubation, the reaction was stopped by adding 0.5 mL of 1 M perchloric acid. The absorbance was recorded at 436 nm using a spectrophotometer, and the H_2_O_2_ content was determined using a standard curve [67].

#### 4.4.3. Electrolyte Leakage (EL)

Leaf samples (0.1 g) were cut into pieces and rinsed with deionized water before being transferred into Falcon tubes containing 10 mL of deionized water. The samples were incubated in a water bath at 32 °C for 2 h, after which the electrical conductivity of the solution was measured (EC_1_). Subsequently, the samples were heated in a boiling water bath at 100 °C for 20 min to completely kill the tissues and release all electrolytes, and the final electrical conductivity was measured (EC_2_). Both electrical conductivity measurements were performed at the same temperature. Electrolyte leakage (EL) was calculated according to the following formula [68]:

EL = (EC_1_/EC_2_) × 100 (2)

#### 4.4.4. Protein Content

Total soluble protein content was determined following the Bradford method using Bovine Serum Albumin (BSA) as a standard [69]. Fresh plant tissue (0.5 g) was homogenized in 3 mL of 0.05 M sodium phosphate (Na-P) buffer (pH 7.8) containing 1 mM EDTA-Na_2_ and centrifuged at 13,000 rpm for 15 min at 4 °C. An aliquot of the supernatant (100 µL) was mixed with 5 mL of Bradford reagent (prepared with Coomassie Brilliant Blue G-250, 95% ethanol, and orthophosphoric acid). After vortexing and incubating for 5 min, the absorbance was measured against a blank at 595 nm using a spectrophotometer. Protein concentrations (mg g^−1^) were calculated using the BSA standard curve. The resulting protein values were used to calculate the specific activity of each enzyme [67].

#### 4.4.5. Catalase Activity (CAT)

Catalase activity was determined by measuring the decomposition of H_2_O_2_ at 240 nm. One enzyme unit was defined as the amount of enzyme that consumes 1 µmol of H_2_O_2_ per minute. CAT activity was expressed as enzyme units per milligram of protein (µ mg^−1^ protein) [70].

#### 4.4.6. Peroxidase Activity (POX)

Tissue samples were homogenized with 2 mL of 0.05 M sodium acetate buffer (pH 6.5). Peroxidase activity was then determined by measuring the change in absorbance at 300 nm for 120 s using a reaction mixture containing 0.05 M sodium acetate buffer (pH 6.5), 0.1 M pyrogallol, and 0.09 M H_2_O_2_ [71].

#### 4.4.7. Ascorbate Peroxidase Activity (APX)

APX activity was determined by monitoring the oxidation of ascorbate at 290 nm. The reaction mixture consisted of 50 mM Na-P buffer (pH 7.0), 0.5 mM L-ascorbate, 0.1 mM EDTA-Na_2_, and 1.2 mM H_2_O_2_. The reaction was initiated by the addition of the enzyme extract, and the decrease in absorbance at 290 nm was monitored for 2 min. APX activity was calculated using an extinction coefficient of 2.8 mM^−1^ cm^−1^. One unit of APX activity was defined as the amount of enzyme required to oxidize 1 μmol of ascorbate per minute under the assay conditions [72].

#### 4.4.8. Glutathione Reductase Activity (GR)

GR activity was determined by monitoring the NADPH-dependent reduction in oxidized glutathione (GSSG) at 340 nm for 3 min. GR activity was calculated using an extinction coefficient of 6.2 mM^−1^ m^−1^. GR activity was expressed as µ mg^−1^ protein, with one U corresponding to the amount of enzyme required to reduce 1 μmol of GSSG per minute under the assay conditions [73].

### 4.5. Determination of Isoenzyme Profiles

All extraction steps were performed at 4 °C. Leaf tissue (0.5 g) was ground to a fine powder in liquid nitrogen and homogenized in 1.5 mL of extraction buffer containing 50 mM Tris–HCl (pH 7.8), 0.1 mM EDTA, 0.2% (*w*/*v*) Triton X-100, 1 mM PMSF and 2% (*w*/*v*) PVP. Homogenates were centrifuged at 14,000× *g* for 15 min, and the supernatants were used for protein and enzyme analyses. Protein concentration was determined by Bradford [69] using BSA as standard. Equal amounts of protein (10 µg per lane) were separated by non-denaturing PAGE according to Laemmli [74], omitting SDS, using a 4.5% stacking gel and a 12.5% separating gel at a constant current of 120 mA at 4 °C. For SOD activity staining, gels were incubated in a solution containing riboflavin and nitro blue tetrazolium (NBT) with gentle shaking in the dark for 60 min [75]. For CAT isoenzyme separation, a 7.5% resolving gel was used; the gels were first soaked in a 0.01% H_2_O_2_ solution for 5 min, followed by staining in a solution containing 1% FeCl_3_ and 1% K_3_Fe(CN)_6_ for 2 min. Upon visualization of the distinct bands, the gels were rinsed with deionized water [76]. Gels were photographed with a *Vilber Lourmat Quantum ST5* gel imaging system, and band intensities were quantified with Bio-Capt software (version 11.0.2.0) after background subtraction and expressed as relative band intensity (arbitrary densitometric units). Three independent gels, each from an independent biological replicate, were analyzed (*n* = 3).

### 4.6. Histochemical Analysis

For histochemical analyses, the first 5 cm of leaves were excised from seedlings, with three independent biological replicates performed for each group.

#### 4.6.1. Superoxide Anion (O_2_^•−^) Accumulation

Superoxide anion (O_2_^•−^) accumulation was visualized using nitroblue tetrazolium (NBT) staining. Leaf samples were immersed in an NBT solution and incubated in the dark at room temperature for 24 h. Following incubation, chlorophyll was removed by boiling the leaves in an acetic acid/ethanol (1:3, *v*/*v*) solution at 95 °C for 10 min. The samples were subsequently placed in 60% glycerol. O_2_^•−^ accumulation was detected as dark blue formazan precipitates resulting from the reaction between NBT and O_2_^•−^. The stained leaves were photographed against a contrasting background for visualization [77].

#### 4.6.2. Hydrogen Peroxide (H_2_O_2_) Accumulation

Leaf samples were incubated in a 1 mg mL^−1^ 3,3′-diaminobenzidine (DAB) solution at 25 °C for 12 h. Following incubation, chlorophyll was cleared by immersing the leaves in a boiling acetic acid/ethanol (1:3, *v*/*v*) solution for 15 min. The samples were subsequently placed in 60% glycerol. H_2_O_2_ accumulation was visualized as reddish-brown spots formed by the reaction between DAB and endogenous H_2_O_2_, and the stained leaves were photographed against a contrasting background [77].

### 4.7. Statistical Analysis

Data were analyzed using SPSS 27 statistical software. A three-way analysis of variance (ANOVA) was performed to evaluate the main effects of variety, water regime, and chemical treatment, as well as their interaction effects (Variety × Water regime × Treatment). Post hoc pairwise comparisons among treatment means were determined using Tukey’s Honestly Significant Difference (HSD) test at *p* < 0.05. Data are presented as means ± standard error (SE), and different lowercase letters indicate statistically significant differences among treatments. In addition, to assess the relationships among physiological and biochemical variables in wheat varieties under drought stress and exogenous treatments, Principal Component Analysis (PCA) and Pearson correlation analysis were performed using Python 3.10.0.

## 5. Conclusions

The present study showed that drought stress induced significant physiological and biochemical alterations in wheat varieties. Exogenous SNP and EBL treatments partially alleviated some adverse effects of drought stress, potentially through modulation of antioxidant defense responses and ROS accumulation. Combined SNP + EBL treatment appeared to provide complementary protective effects, particularly in the drought-sensitive variety. These findings suggest that NO and BR treatments may contribute to improving physiological and biochemical responses associated with drought tolerance in wheat varieties.

## Figures and Tables

**Table 1:** Experimental treatments and concentrations of SNP and EBL-treated to wheat seedlings under control and drought conditions.

TREATMENT GROUP		CONCENTRATION
1	CONTROL	0 µM/mg L^−1^
2	SNP	200 µM
3	EBL	0.05 mg L^−1^
4	SNP + EBL	200 µM + 0.05 mg L^−1^
5	PEG	0 µM/mg L^−1^
6	SNP + PEG	200 µM
7	EBL + PEG	0.05 mg L^−1^
8	SNP + EBL + PEG	200 µM + 0.05 mg L^−1^
9	WATER SCARCITY	0 µM/mg L^−1^
10	SNP + WATER SCARCITY	200 µM
11	EBL + WATER SCARCITY	0.05 mg L^−1^
12	SNP + EBL + WATER SCARCITY	200 µM + 0.05 mg L^−1^

SNP: Sodium Nitroprusside, EBL: 24 Epibrassinolide, PEG: Polyethylene glycol.

## Data Availability

The original contributions presented in the study are included in the article; further inquiries can be directed to the corresponding author.
